# Toward Personalized Medicine for HIV/AIDS

**DOI:** 10.33696/AIDS.3.020

**Published:** 2021

**Authors:** Carla M. Kumbale, Eberhard O. Voit

**Affiliations:** The Wallace H. Coulter Department of Biomedical Engineering at Georgia Tech and Emory University, 950 Atlantic Drive, Suite 2115, Atlanta, Georgia 30332-2000, USA

In 2019, approximately 38 million people were infected with HIV worldwide [1]. Although there is still no cure that successfully eradicates the disease, combination antiretroviral therapy (cART) has improved to a point where undetectable viral loads have become achievable and HIV carriers often live almost normal lives with very substantially increased average life expectancies compared to historical data [2,3]. While the disease in many cases becomes chronic, the specifics of its progression in an individual may diverge dramatically from the average and thus manifest differently in each affected person. This variability is of concern, causing one to wonder whether an average treatment regimen is optimal for a given individual.

If the viral infection were untreatable, or if there were just one treatment, addressing this concern would be moot. However, the options within cART have dramatically changed the landscape of treating HIV/AIDS [4,5]. More than two dozen antiretroviral drugs have been approved by the FDA and are in current use [6]. They fall into several classes that interfere with different aspects of virus replication or through different mechanisms [4,7]: (1) specific CD4-directed post-attachment inhibitors bind to chemokines coreceptors, thereby preventing HIV from attaching to and entering host cells; (2) chemokine receptor antagonists (CRAs) selectively block interactions between the human CCR5 receptor and the HIV-1 gp120 protein which, in turn, prevents HIV entry into cells; (3) fusion inhibitors (FIs) disrupt HIV binding and, ultimately, fusion with host cells; (4) the transcription of viral RNA into double-stranded DNA can be prevented with nucleoside or nucleotide reverse transcriptase inhibitors (NRTIs); (5) targeting the same process, the activity of the key enzyme HIV-1 reverse transcriptase may be reduced or blocked with non-nucleotide reverse transcriptase inhibitors (NNRTIs); (6) integrase inhibitors (IIs) hinder the transport and attachment of pro-viral DNA to host-cell chromosomes; (7) HIV replication and the formation of mature, infectious viral particles can be prevented with protease inhibitors (PIs); (8) pharmacokinetic enhancers do not directly interfere with viral replication but rather boost the concentration of antiretrovirals in the blood to make them more effective. Numerous variations of cART have been approved. The initial therapy usually starts with a combination of three antiretrovirals, including two NRTIs plus an NNRTI, or two NRTIs plus a protease inhibitor. The use of four antiretrovirals was shown not to improve outcomes over a combination of three compounds [8].

One challenge for any therapy, including cART, is HIV latency, a phenomenon where the virus persists mostly in resting memory CD4+ T cells, as well as possibly in other cell types at different locations within the body, and can re-emerge when treatment is discontinued [9]. While these latency mechanisms are not fully understood, it appears that the viral integration site and pro-viral orientation, as well as genomic architecture and stochastic gene expression, are contributing factors [10]. One treatment strategy has been the reactivation and then depletion (“shock and kill”) of virus in the latent reservoirs [11], while another approach has been an attempt to manipulate the signaling pathways that are essential for the establishment of latency [12]. However, a complete eradication has so far not been achieved on a regular basis [9,12] and cART is to be continued throughout a carrier’s life.

The variations in cART regimens, made possible by the different approved antiretroviral drugs, theoretically provide very many alternative therapeutic options, especially if different dosing regimens are feasible. This variability suggests that one might envision customization of treatment to specific individuals or groups of individuals [13]. While the combinatorial choices among many alternatives are certainly very welcome, the question arises how one might achieve the best match between an individual and an optimal, custom-tailored treatment regimen, which includes occasional changes in drug combinations that might become necessary due to the development of drug resistance or side effects that are difficult to tolerate. In fact, it is expected that not all combination therapies are similarly efficacious for all patients, may cause severe side effects, or even fail. Designing optimally effective, individualized treatment regimens with the best morbidity profiles for each patient will be challenging with experimental and clinical approaches, but appears to be well-suited for exploration within the emerging field of personalized medicine.

The emerging concept of personalized medicine and its counterpart of predictive health proposes a widening of the scope of potential treatments with the goal of allowing more flexible matching of an individual’s case with a specific treatment and dosing regimen [14,15]. This possible expansion in scope is not without risks, as substantive deviations from a standard treatment may be deleterious and possibly life-threatening. Moreover, non-standard approaches of care may not be reimbursed by insurance providers and could expose the physician to litigation.

Thus, before systemic deviations from standard treatment procedures are to be considered for a specific individual, one must ask why this individual could or should possibly respond better to a customized treatment than to the standard of care. Many answers could be given. An important but implicit reason is associated with the fact that drug treatments are based on population averages. These averages derive at first from animal studies, where sufficiently large samples of mice, rats, or other mammals were exposed to the drug in order to study likely side effects, and where additional samples were later used to assess efficacy against the disease. The same scenario is repeated, *mutatis mutandis*, in clinical trials. The conceptual rationale for this procedure is absolutely valid, namely, to evaluate whether a treatment can alleviate disease in the study population without causing harm or undue side effects. The potential opportunity to improve on the outcome of this traditional technique comes from the possibility that a given individual may have a genetic and physiological make-up that could benefit from a drug or high dosing regimen while being resistant or tolerant to severe side effects.

In our recent article [16], *Dynamical Systems Approaches to Personalized Medicine*, we discuss the emerging role of computational systems biology for the implementation and practical use of personalized medicine. We motivate our analysis with a beautiful illustration, namely a study on acute lymphoblastic leukemia (ALL) in children, for which chemotherapy can have a long-term disease-free survival rate of about 90%. At issue are the remaining 10% of children, many of whom in the past had severe if not fatal side effects from this very treatment [17]. Instead of abandoning the obvious potential of the specific chemotherapy, clinicians and physician-scientists designed a transcriptomic study on leukemia cells from cohorts of pre-treatment patients, which revealed between 20 and 45 differentially expressed genes that were associated with single-drug resistance or cross-resistance [18]. By testing children with respect to these prognostic transcriptomic signatures, it became possible to differentiate responders from non-responders to the treatment with high reliability [19]. Several other examples of risk stratification have been published that were based on specific molecular aspects of a targeted cohort (see, e.g., [17]).

While it is quite evident and unsurprising that personalized medicine has high potential, the question arises of what it will take to make this emerging approach reality. The answer consists of two complementary components. The first is the availability of sufficient personalized information, while the second is a set of robust methods for analysis, interpretation, and prediction.

Databases of personalized information require collecting large amounts of pertinent data from individuals through imaging, -omics profiling, and the measurement of physiological or molecular biomarkers that could be generic or disease-specific, possibly in combination with one of the many new health monitoring devices that have become available in recent times [20–22].

While of undisputed importance, this collection of data is a necessary but insufficient prerequisite. In fact, it does not even always suffice to compare an individual’s “molecular profile” with that of the general population, because many differences may be physiologically neutral, at least with respect to the disease at hand. Instead, the next critical step beyond simple comparisons may proceed into two directions.

First, once all feasible molecular information of an individual is collected, computational methods of statistical machine learning and artificial intelligence may be able to extract patterns from the data that are associated with the disease [23]. This approach is very powerful if an entire cohort of individuals is tested, some of whom are healthy and some diseased. Specifically coded and trained computer algorithms then are often capable of detecting complex patterns among the data that extract distinguishing markers of disease.

Second, if a comprehensive computational model of the disease exists, the molecular profile of the individual can be represented within this model structure [20,22,24]. This computational approach proceeds as follows: The disease model is composed of a system of equations; usually, these are differential equations [25,26]. These equations always contain a typically large set of numerical parameters that determine critical features of the phenomenon, such as the half-life of CD4 cells, the speed at which the disease progresses, or the rate at which the immune system reduces the viral load. Initially, these parameters are determined from averages obtained from clinical or epidemiological measurements and animal experiments [27]. The personal embedding is accomplished when as many of these “average” model parameters are replaced with parameter values corresponding to personalized measurements taken from the individual in question. The result is a disease model adjusted to the specific molecular properties of a single individual [16,24]. It is obviously not possible to measure every single parameter in the same individual, and values of parameters that cannot be acquired through measurements are—by necessity—retained as the population averages previously obtained; the number of these parameters should gradually be decreased. This method to personalized medicine is illustrated in Figure 1. Once a disease model has been calibrated with an individual’s parameter values, it is comparatively easy to simulate health and disease, explore treatment options, and forecast future health trajectories.

This model-based approach, utilizing personalized data and their computational analysis within something like an *ex vivo* context, actually offers more than personalized health predictions, namely the ability to understand why a disease may manifest differently among individuals and why certain drugs used to treat the disease may not be similarly effective in all patients.

It is clear that we are far from perfect personal molecular testing and simulation-based, individualized predictions, but various scientific communities have taken the first critical steps in this direction. The amounts of data achievable in a standard molecular biology lab have grown exponentially both in quantity and also in quality, and data on single individuals or even single cells are becoming more commonplace. At the same time, computer scientists have multiplied computational power and made this power available to non-experts, while researchers in biomathematics and the nascent field of computational systems biology have made great strides toward constructing “average” models of healthy and diseased subsystems of the human body.

A few examples in the context of HIV/AIDS offer a portrayal of the nascent state of the art. A review by Prague *et al*. [28] highlights the importance of mechanistic models toward achieving more personalized HIV therapeutics. The authors specifically discuss how models consisting of ordinary differential equations have already been used to optimize therapeutics for HIV-infected individuals. As an illustration, they refer to a pioneering modeling approach proposed by Wei *et al*. [29], who computationally assessed the fundamental phenomenon of HIV rapidly mutating, which in turn constitutes the rationale for combining antiretroviral drugs with different mechanisms of action.

Prague and colleagues [28] do not hide the major obstacle inherent in the use of these dynamical models, namely, the difficulty of estimating model parameters [30]. While parameter optimization is a true challenge, trends throughout the past few decades leave little doubt that this bottleneck will gradually become less of an issue. At the same time, the rewards of the modeling approach will be plentiful, because it will eventually become a routine analysis to test new drugs or drug combinations not only on averaged mathematical models for populations but also on personalized models of individuals. Once we approach this point, these models will become invaluable tools for testing a wide spectrum of hypotheses into varying biomarkers and biological mechanisms and for allowing objective comparisons of therapeutic regimens of personalized HIV treatment.

Of course, HIV/AIDS is a complex disease. Lengauer *et al*. [31] singled out the phenomenon of evolving drug resistance. Drug cocktails in cART are designed to prevent (or at least slow down) drug resistance, but once a cocktail fails, predicting a new, efficacious drug combination is difficult to determine because many different drugs are available for HIV, and an appropriate dose must be specified for each component of the new cocktail. Clearly, a reliable, personalized computational HIV simulator would be a welcome addition to the physician’s treatment repertoire.

Mu *et al*. [3] discuss different strategies for improving personalized medicine in the context of HIV. They note, in particular, that therapeutic drug monitoring (TDM) is currently not part of standard care, but should be considered especially for patients at elevated risk who, for instance, are pregnant, do not respond to therapy, or suffer from suspected drug-drug or drug-food interactions. At present, insufficient cost-effectiveness is the main reason not to use TDM as a part of routine therapy, but Mu *et al*. argue convincingly that TDM has been effectively utilized retrospectively to determine why certain individuals had not experienced an expected therapeutic response. Thus, instead of a retrospective application, they propose TDM could enable the possibility of prospectively “just-in-time” determining which patients would benefit from a certain treatment. The authors support this assertion with an example where individuals, who had been administered the drug combination of lopinavir with ritonavir, were found to be significantly less likely to experience treatment failure if they had certain MDR1 polymorphisms [32]. Thus, prospective testing of these polymorphisms might be able to suggest with some reliability which patients would benefit from the combination of lopinavir and ritonavir.

These studies support the prospect that HIV infections are a fertile ground for exploring personalized treatments. Much still needs to be done toward a general strategy that can reliably predict which combination therapy would be best suited for a given individual. The challenge now is developing a deeper understanding of the exact mechanism with which HIV eventually outfoxes the human immune system and how we might help the body control the virus’ antigenic variation.

Given the complexity of the host-virus system, it appears that forays into personalized HIV treatments might greatly benefit from computational support. Mathematical models are in principle able to integrate hundreds of processes in a functional and meaningful manner, and their main limitation is more and more the availability of crisp, quantitative data. Once a personalized HIV model is established for an individual, it will become possible to make short-term predictions and correct the model based on comparisons with actual observations after a short period of time, if not even instantaneously in an online manner. Models of this type are already being used for glucose imbalances caused by diabetes, and given time, they might assist individuals with HIV on a daily basis [33].

## Figures and Tables

**Figure 1:**
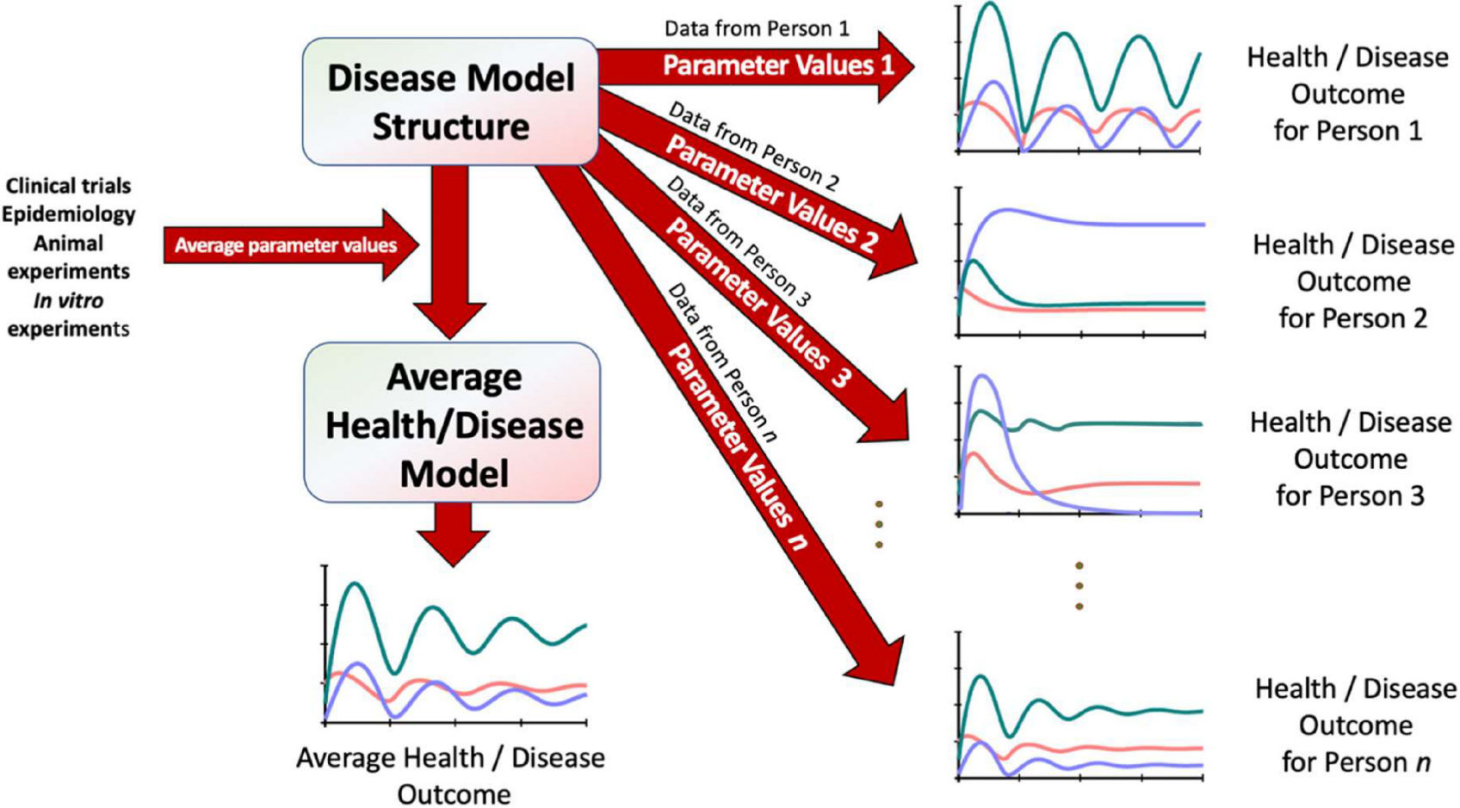
Personalized health and disease modeling uses a model structure that was designed based on general knowledge of a disease, combined with parameter values that come either from clinical trials and animal experiments and ultimately yield an average model, or from individuals, in which case the combined result is a set of personalized models, one for each individual. The individual model responses may be very similar or can be quite different. Adapted from Davis *et al.* [16].
